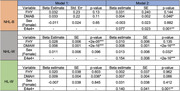# Family history and amyloid PET in white, Black, and Hispanic/Latino, cognitively normal individuals

**DOI:** 10.1002/alz.091146

**Published:** 2025-01-09

**Authors:** Phelan Glenn, Katelyn Elizabeth Mooney, Ose Kadiri, Talia L Robinson, Rebecca E Amariglio, Reisa A Sperling, Kacie Deters

**Affiliations:** ^1^ University of California, Los Angeles, Los Angeles, CA USA; ^2^ University of California, Los Angeles Neuroscience Interdepartmental Program (NSIDP), Los Angeles, CA USA; ^3^ Massachusetts General Hospital, Boston, MA USA; ^4^ Center for Alzheimer Research and Treatment, Brigham and Women’s Hospital, Harvard Medical School, Boston, MA USA; ^5^ Brigham and Women's Hospital, Harvard Medical School, Boston, MA USA; ^6^ University of California, Los Angeles Integrative Biology and Physiology (IBP), Los Angeles, CA USA

## Abstract

**Background:**

Family history (FH) of AD increases risk for sporadic AD, and has been associated with increased amyloid accumulation. Most of these studies were done in non‐Hispanic white groups, limiting the generalizability of these findings. The goal of this study was to determine if FH was associated with levels of amyloid in White, Black, and Hispanic/Latino participants.

**Methods:**

This study included cognitively normal individuals screening for the Anti‐Amyloid Treatment in Asymptomatic Alzheimer’s (A4) cohort whom self‐identified as Non‐Hispanic/Latino Black (NHB; N=145), Non‐Hispanic white (NHW; N=3766), or Hispanic/Latino white (HL; N=107). The effect of FH (father, mother or both, vs none) on amyloid PET SUVr was assessed using linear regression analysis within each ethnic and racialized group. Age and sex were used as covariates. This analysis was repeated adding APOE e4 allele as a moderator.

**Results:**

NHB participants were more likely to be female (71.7%), had the lowest FH rate (56.6%), and were less likely to be amyloid positive (20%) compared to HL participants (female=64.5%; FH=62.6%; amyloid+=27.1%) and NHW participants (female=59.7%; FH=66.2%; amyloid+=30.2%). Having a FH of AD was associated with higher levels of amyloid SUVr (β=0.028, p=2e‐05) in NHW participants only (Table). This effect became non‐significant when APOE e4 was included in the model.

**Conclusions:**

FH was associated with higher levels of amyloid SUVr in White participants only, but not in the presence of APOE e4. For Black and Hispanic participants, FH was not associated with levels of amyloid. These findings support previous findings in which risk factors for AD are not shared equally across different ethnic and racialized groups.